# The Fate of Patients with Solitary Pulmonary Nodules: Clinical Management and Radiation Exposure Associated

**DOI:** 10.1371/journal.pone.0158458

**Published:** 2016-07-08

**Authors:** Blanca Lumbreras, José Vilar, Isabel González-Álvarez, Noemí Gómez-Sáez, María L. Domingo, María F. Lorente, María Pastor-Valero, Ildefonso Hernández-Aguado

**Affiliations:** 1 Public Health Department, Miguel Hernández University, Alicante, Spain; 2 CIBER en Epidemiología y Salud Pública, Madrid, Spain; 3 Radiodiagnostic Department, Peset Hospital, Valencia, Spain; 4 Radiodiagnostic Department, San Juan Hospital, Alicante, Spain; University of North Carolina School of Medicine, UNITED STATES

## Abstract

**Background:**

The appropriate management of the large number of lung nodules detected during the course of routine medical care presents a challenge. We aimed to evaluate the usual clinical practice in solitary pulmonary nodule (SPN) management and associated radiation exposure.

**Methods:**

We examined 893 radiology reports of consecutive patients undergoing chest computed tomography (CT) and radiography at two public hospitals in Spain. Information on diagnostic procedures from SPN detection and lung cancer diagnosis was collected prospectively for 18 months.

**Results:**

More than 20% of patients with SPN detected on either chest radiograph (19.8%) or CT (26.1%) underwent no additional interventions and none developed lung cancer (100% negative predictive value). 346 (72.0%) patients with SPN detected on chest radiograph and 254 (61.5%) patients with SPN detected on CT had additional diagnostic tests and were not diagnosed with lung cancer. In patients undergoing follow-up imaging for SPNs detected on CT median number of additional imaging tests was 3.5 and the mean cumulative effective dose was 24.4 mSv; for those detected on chest radiograph the median number of additional imaging tests was 2.8 and the mean cumulative effective dose was 10.3 mSv.

**Conclusions:**

Patients who did not have additional interventions were not diagnosed of lung cancer. There was an excessive amount of interventions in a high percentage of patients presenting SPN, which was associated with an excess of radiation exposure.

## Introduction

A large number of lung nodules are detected during the course of routine medical care and their appropriate management presents a challenge [1]. There is sufficient evidence of the negative consequences stemming from poorly targeted investigations [2–7]. However, there is no evidence about the exposure to radiation during the management of a pulmonary nodule, defined as a pulmonary opacity up to 30 millimetres in diameter [8].

Given the natural history of lung cancer, clinicians tend to adopt a proactive attitude performing clinical interventions that are often unnecessary [9]. Recently, the British Thoracic Society (BTS) has published guidelines for the management of pulmonary nodules [10]. These guidelines present the most up to date and evidence based recommendations for follow up, although most of these recommendations are classified as Grade C or D. Moreover, in contrast with Fleischner recommendations [11] and in agreement with the latest information from the NELSON trial, [12] they do not recommend follow-up for people with nodules <5 mm in maximum diameter.

A recently published retrospective study on 300 adults with pulmonary nodules from 15 Veterans Affairs hospitals reveals that pulmonary nodule evaluation is often inconsistent with the Fleischner Society guidelines [13]. Moreover, there is some data that shows marked variability in the management of SPN detected on CT in usual clinical practice [14]. A recent study focused on patients with intermediate-sized nodules who were referred to community-based pulmonologists, showed that 44% of low-risk patients underwent one or more invasive procedures for a benign nodule [15].

New guidelines in the SPN management have been published. However, given that the majority of research studies involving pulmonary nodules are in screened populations [16–19], there is a need to evaluate the management of SPNs detected on imaging studies ordered for a number of reasons in the general clinical setting. The evaluation of the impact of these different management strategies carried out in patients with SPN on the diagnosis of lung cancer in a routine setting would be an essential guide for clinicians. In addition, the measurement of the radiation exposure associated with different clinical pathways could be useful when making clinical decisions.

Therefore, the aim of this prospective study was to describe the management of SPN detected on both chest radiograph and CT scans performed in routine practice including variables associated with management decisions, final diagnoses and total radiation exposure.

## Material and Methods

### Study subjects

During the years 2010 and 2011, all patients ≥ 35 years free of lung cancer referred for thoracic imaging evaluation in two general public hospitals in the Valencian Community (Spain) were prospectively collected from the radiological electronic record (a total of 25,529 patients). Eight expert radiologists working in the radiologist departments at the time in both hospitals determined the presence of SPN in the thoracic study of these patients (a total of 893 (3.5%) out of 25,529 examined) [20]. Information on diagnostic procedures from SPN discovery and lung cancer diagnosis was collected prospectively for 18 months. In these 893 patients with SPN detected, the risk of developing lung cancer in an 18 month follow-up period was nearly 10% [21]. The target population of the present study consisted of the residents of the catchment area of the participating public hospitals: San Juan Hospital and Dr Peset Hospital, with a catchment population of 234,424 and 377,780 people, respectively. The two hospitals belong to the National Health Care System and are referral hospitals for all individuals living in the same geographical area. The majority of the population in these areas uses the National Health Care System as the main medical service.

Institutional Review Board approval (University Miguel Hernandez Committee Ref DSP-BLL-001-10) was obtained. Given the study uses only routine data and no additional interventions, informed consent was not sought from the patients and this was approved by the IRB. Patient information was anonymized and participants’ identification code numbers were de-identified by replacing the original code number with a new random code number.

### Data collection

Patients were classified into two groups according to the initial imaging exam on which the SPN was identified: chest radiograph or CT.

#### Detection and description of the SPN

Eight chest expert radiologists (all of them with more than 10 years of experience) determined the presence of SPN in thoracic studies of the 25,529 patients initially included. We limited our study to nodules between and 30 millimeters [8]. Intrapulmonary lymph nodes and pseudolesions, when detected, were excluded from our study.

Chest radiographs were obtained with the standard technique in digital format (CR Philips). The CT technique varied according to the study that was being performed. CT imaging tests were obtained with slice thicknesses of 3mm or less (2 mm, 1.5 and 1.25) according to the different clinical situations and the equipment used, 120 KvP and variable mAs. The nodules were measured using calipers in the PACS workstations in their largest diameter in the posterior anterior and lateral radiograph. CT lung window settings (1550/-600) were used to measure nodule size in the largest diameter. Mediastinal window settings (350/50) were also used to further detect calcification or fat (<40 Housnfield units) within the nodule.

The radiologists described nodule characteristics in a predesigned form consisting of: a) size, in mms, and also expressed as mean (sd) in diameter; b) nodule shape, smooth or irregular (lobular or spiculated); c) location, and d) for those patients who underwent a CT, nodule consistency (solid, partly solid, ground glass, calcification or not specified). As we previously reported [20], for the evaluation of inter-observer agreement in particular aspects such as nodule size, shape or consistency, the first 300 tests included were evaluated independently by the 8 radiologists. For the intra-observer agreement of these characteristics, the radiologists re-evaluated 200 studies at least 6 months after the first report. In case of discordance, the consensus was established by the 2 radiologists with more experience (IGA and JV).

#### Patients’ characteristics

Selected clinical and demographic variables were gathered from the medical records for all patients: type of test performed; department that ordered the test; care setting; reason for test and patient characteristics. Moreover, we collected from the medical records: smoking status, previous neoplasm, presence of a respiratory disease and respiratory symptoms.

### Follow-up

Prospective follow-up during 18 months was conducted in all 893 patients through the review of their medical records, including the ascertainment of lung cancer diagnosis and the specific cause of death (if they occurred).

All the additional interventions carried out were collected on a pre-established form. Based on Fleischner Society Criteria [11] of a three-month interval for follow-up imaging, immediate intervention was defined as any study performed within a three month window following SPN identification and additional imaging was defined as any imaging study performed at or beyond three months form SPN identification.

In those patients who underwent CT as an additional or immediate intervention, we distinguished between those patients who underwent 1 CT (labelled in the results section as ‘CT’) and those who underwent 1 or more follow-up CT three months after the previous test was carried out (labelled in the results section as ‘CT surveillance’). A diagnosis of lung cancer was confirmed by cytopathological evaluation of needle-aspiration biopsy samples or histopathologic examination of surgically resected specimens [22]. Tissue was always obtained from the primary lung lesions.

We estimated the cumulative effective dose of diagnostic procedures in the study population according to previous evidence [23]: chest radiograph (0.1 mSv), chest CT (7 mSv), PET/CT (25 mSv). We compared the effective doses received in those patients who ascertained a diagnosis of lung cancer and those who did not.

### Statistical analysis

All data was computerized and checked to discard errors. Statistical precision was determined through the calculation of 95% CI. All analyses were carried out with the statistical programme Stata 8 (Stata Corp., College Station, Texas, USA).

A descriptive analysis was performed to estimate the proportions of the patients and nodule characteristics according to the immediate next step in management (immediate intervention vs additional imaging studies or no further work upnot further working). A descriptive analysis was carried out using frequency distribution or median and interquartile range (IQR) when appropriate. The age was transformed in quartiles because the equal variance and normal distribution was rejected. Nodule diameter was categorized according to Fleischner recommendations.

## Results

**Fig 1:**
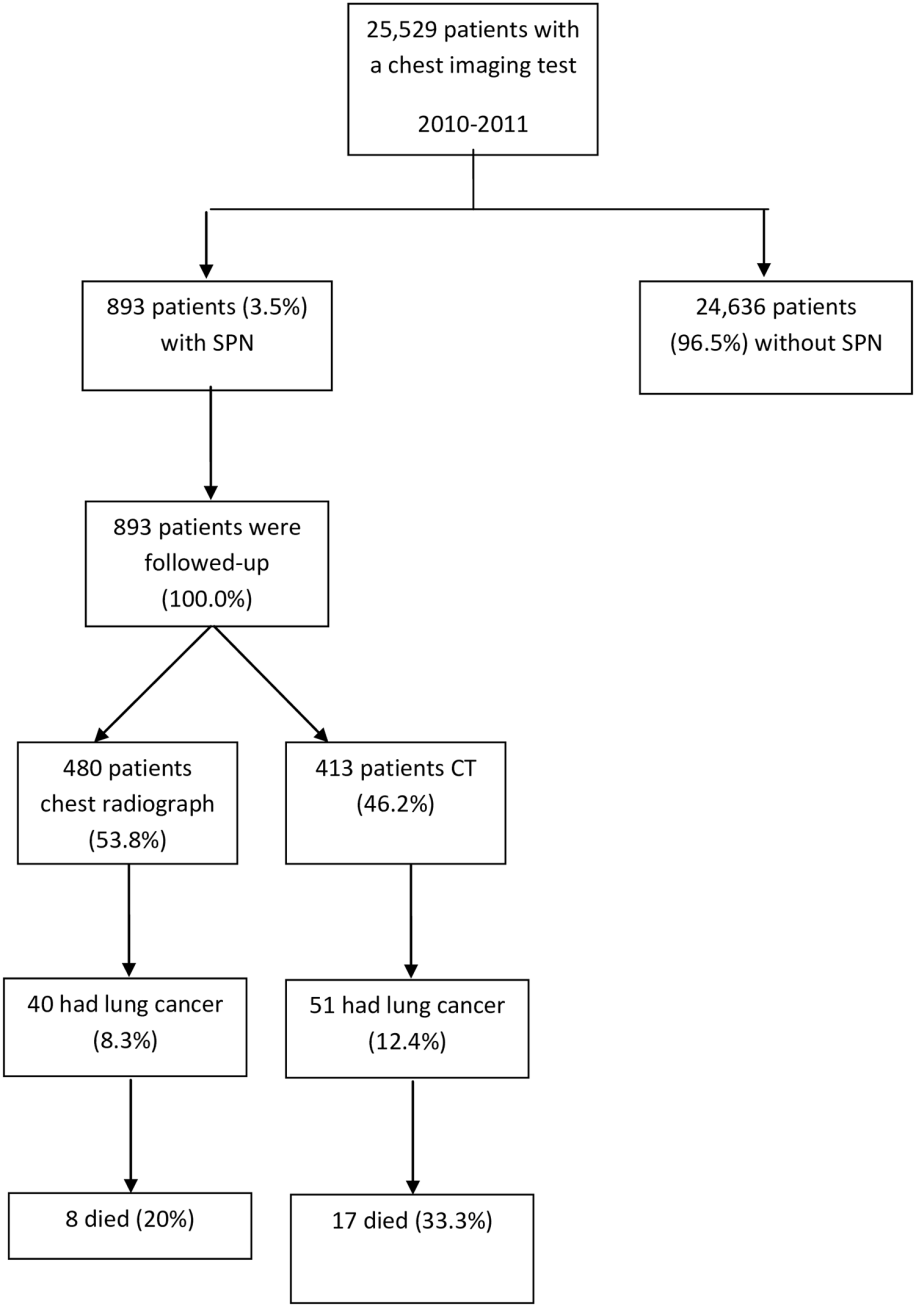
Flow diagram showing the follow-up of the study participants.

Fig 1 illustrates the lung cancer incidence and specific mortality in the 893 patients with SPN during the 18 months of follow-up. Clinicians decided to perform immediate interventions in 410 patients (45.9%); follow-up in 280 cases (31.3%) and in 203 patients (22.7%) clinicians decided not test further. The diverse management strategies carried out in patients having SPN for both imaging tests and the detailed description of the diagnostic pathway is given in S1 and S2 Text, S1 and S2 Figs. In our study, there were no patients who underwent invasive procedures (including CT guided biopsy, bronchoscopy, or surgery) as the immediate next test following SPN identification. In brief:

### Diagnostic pathway (Table 1)

**Table 1:** Description of patient’s characteristics according to the clinical management strategy after the detection of a SPN (for chest radiograph and CT) and lung cancer diagnosis after 18 months of follow-up*.

VARIABLES	Chest radiograph					CT				
N (%)										
(95% CI)										
	Total 480 (lung cancer 40; 8.3%)	No further testing 95 (19.8%) (lung cancer 0)	Follow-up 84 (17.5%) (lung cancer 4; 4.8%)	Immediate interventions 301 (62.7%) (lung cancer 36; 11.9%)	*p-value*	Total 413 (lung cancer 51; 12.4%)	No further testing 108 (26.2%) (lung cancer 0)	Follow-up 196 (47.5%) (lung cancer 11; 5.6%)	Immediate interventions 109 (26.4%) (lung cancer 40; 36.7%)	*p-value*
**Gender**					*0*.*000*					*0*.*191*
Male	285	51 (17.9)	36 (12.6) **(2; 5.5)**	198 (69.5) **27(13.6)**		261	72 (27.6)	115(44.1) **(9; 7.8)**	74 (28.4)**(34; 45.9)**	
Female	195	44 (22.6)	48 (24.6) **(2; 4.2)**	103 (52.8)**(9; 8.7)**		152	36 (23.7)	81 (53.3)**(2; 2.5)**	35 (23.0)**(6; 17.1)**	
**Age in years**					*0*.*056*					*0*.*055*
< 50	76	18 (23.7)	7 (9.2) **(1;14.3)**	51 (67.1)**(2; 3.9)**		50	16 (32.0)	22 (44.0)**(1; 4.5)**	12 (24.0)**(5; 41.7)**	
50–59	86	12 (14.0)	22 (25.6)**(2; 9.1)**	52 (60.5)**(10; 19.2)**		87	12 (13.8)	45 (51.7)**(2; 4.4)**	30 (34.5)**(8; 26.7)**	
60–69	132	20 (15.2)	23 (17.4)(0)	89 (67.4)**(11; 12.4)**		126	31 (24.6)	64 (50.8)**(4; 6.3)**	31 (24.6) **(12; 38.7)**	
≥70	186	45 (24.2)	32 (17.2)**(1; 3.1)**	109 (58.6)**(13; 11.9)**		150	49 (32.7)	65 (43.3)**(4; 6.2)**	36 (24.0)**(15; 31.7)**	
**Reason for requesting imaging test**					*0*.*696*					*0*.*662*
Preoperative	86	21 (24.4)	17 (19.8)**(0)**	48 (55.8)**(10; 20.8)**		80	21 (26.3)	34 (42.5)**(1; 2.9)**	25 (31.3)**(11; 44.0)**	
Respiratory	135	23 (17.0)	25 (18.5)**(1; 4.0)**	87 (64.4)**(9; 10.3)**		85	28 (32.9)	37 (43.5)**(1; 2.7)**	20 (23.5)**(9; 45.0)**	
Non-respiratory	102	18 (17.7)	20 (19.6)**(2; 10.0)**	64 (62.8) **(9;14.1)**		108	30 (27.8)	51 (47.2)**(6; 11.7)**	27 (25.0)**(9; 33.3)**	
Extra pulmonary neoplasm	50	8 (16.0)	7 (14.0)**(0)**	35 (70.0)**(3; 8.6)**		51	10 (19.6)	27 (52.9)**(2; 7.4)**	14 (27.5)**(5; 35.7)**	
Not specified	107	25 (23.4)	15 (14.0)**(1; (6.7)**	67 (62.6)**(5; 7.5)**		89	19 (21.4)	47 (52.8)**(1; 2.1)**	23 (25.8)**(6; 26.1)**	
**Smoking status**					*0*.*462*					*0*.*183*
Never	99	22 (22.2)	18 (18.2)**(0)**	59 (59.6)**(2; 3.5)**		92	28 (30.4)	45 (48.9) **(1; 2.2)**	19 (20.7) **(1; 5.3)**	
Ever	275	49 (17.8)	44(16.0)**(4; 9.1)**	182 (66.2)**(31; 17.0)**		252	60 (23.8)	115 (45.6)**(8; 6.9)**	77 (30.6)**(39;50.6)**	
Not specified	106	24 (22.6)	22 (20.8) **(0)**	60 (56.6)**(3; 5.0)**		69	20 (29.0)	36 (52.2)**(2; 5.6)**	13 (18.8)**(0)**	
**Previous malignancy**					*0*.*066*					*0*.*891*
No	313	70 (22.4)	48 (15.3)**(2; 4.2)**	195 (62.3) **(21; 10.8)**		286	75 (26.2)	135 (47.2)**(7; 5.2)**	76 (26.6)**(28; 36.8)**	
Yes	167	25 (15.0)	36 (21.6)**(2; 5.6)**	106 (63.5) **(15; 14.2)**		126	33 (26.2)	60 (47.6)**(4; 6.7)**	33 (26.2)**(12; 36.4)**	
Not specified	-	-	-	-		1	0 (0.0)	1 **(0)**	0 (0.0)	
**COPD**					*0*.*378*					*0*.*100*
No	361	75 (20.8)	66 (18.3)**(3; 4.5)**	220 (60.9)**(19; 8.6)**		296	80 (27.0)	148 (50.0)**(7; 4.7)**	68 (23.0)**(21; 30.9)**	
Yes	119	20 (16.8)	18 (15.1)**(1; 5.6)**	81 (68.1)**(17; 21.0)**		116	28 (24.1)	47 (40.5)**(4; 8.5)**	41 (35.3)**(19; 46.3)**	
Not specified						1	0 (0.0)	1 (0)	0 (0.0)	
**Respiratory Symptoms**					*0*.*123*					*0*.*695*
No	404	85 (21.0)	73 (18.1)**(3; 4.1)**	246 (60.9)**(31; 12.6)**		345	91 (26.4)	163 (47.2)**(10; 6.1)**	91 (26.4)**(35; 38.5)**	
Yes	52	4 (7.7)	9 (17.3)**(1; 11.1)**	**39 (75.0)(4; 10.3)**		57	14 (24.6)	26 (45.6)**(1; 3.8)**	27 (29.8)**(4; 14.8)**	
Not specified	24	6 (25.0)	2 (8.3)**(0)**	16 (66.7)**(1; 6.3)**		11	3 (27.3)	7 (63.6)**(0)**	1 (9.1)**(1; 100.0)**	

^(^*^)^ Lung cancer cases are shown in bold for each category together with the risk as a percentage.

#### Not tested further

95 (19.8%) out of 480 patients with SPN detected on chest radiograph and 108 (26. 2%) out of 413 patients with SPN detected on CT were not further tested and none of them were diagnosed with lung cancer during the 18 month-follow-up study. Most of them were benign granuloma.

#### Follow-up three months after the detection of SPN

Out of 84 (17.5%) patients with SPN detected on chest radiograph, 35 (41.7%) were followed up with chest radiograph, 48 (57.1%) with CT and 1 (1.2%) with PET/CT. Four (4.8%) patients were diagnosed with lung cancer (median time to diagnosis: 9 months; IQR 7–11 months). Out of 196 (47.5%) patients with SPN detected on CT, 18 (9.2%), were followed up with chest radiograph, 175 (89.3%) with CT (89.3%) and 3 (1.5%) with PET/CT. Eleven (5.6%) patients were diagnosed with lung cancer (median time to diagnosis: 7 months; IQR 6–12 months).

#### Immediate intervention

Out of 301 (62.7%) patients with SPN detected on chest radiograph, 67 (22.3%) had immediate interventions with chest radiograph, 225 (78.4%) with CT and 29 (12.9%)with PET/CT and 36 (11.9%) patients were diagnosed with lung cancer (median time to diagnosis: 2 months; IQR 1–4 months). 109 (26.4%) patients with SPN detected on CT had immediate interventions with chest radiograph (3, 2.7%), CT (20, 18.3%) or PET/CT (47, 43.1%). 40 (36.7%) patients were diagnosed with lung cancer (median time to diagnosis: 0.8 months; IQR 0.3–1.5 months).

### Determinants of diagnostic pathways

According to Table 1, in patients with SPN detected on chest radiograph, men had more frequently immediate interventions than women (198/285, 69.5% and 103/195, 52.8%, respectively; p<0.001).

In both patients with SPN detected on chest radiograph and CT, immediate interventions were more frequent in patients with a spiculated nodule (41/50, 82.0% and 35/67, 52.2%, respectively) (p = 0.027 and p<0.001, respectively). Of those patients who underwent immediate interventions lung cancer was diagnosed in 21 patients with spiculated nodules and 1 patient with a smooth nodule (in patients with SPN initially detected on chest radiograph) and in 20 patients with spiculated nodules and 1 patient with a smooth nodule (in patients with SPN initially detected on CT) (Table 2).

**Table 2:** Description of SPN’s characteristics according to the clinical management strategy after the detection of a SPN (for Chest radiograph and CT) and lung cancer diagnosis after 18 months of follow-up*.

VARIABLES	Chest radiograph					CT				
N (%)										
(95% CI)										
	Total 480 (100%) (40; 8.3%)	No further testing 95 (19.8%) (0)	Follow-up 84 (17.5%) (4; 4.8%)	Immediate interventions 301 (62.7%) (36; 11.9%)	*p-value*	Total 413 (100%) (51; 12.4%)	No further testing 108 (26.2%) (0)	Follow-up 196 (47.5%) (11; 5.6%)	Immediate interventions 109 (26.4%) (40; 36.7%)	*p-value*
**Diameter (mm) (mediam. IQR)**	10 (7–14)	9 (6–15)	10 (6–15)	10 (7–15)	*0*.*101*	8 (5.4–13)	6.4 (5–10)	7 (5–10)	14 (9.7–20)	*0*.*000*
**Localization**					*0*.*645*					*0*.*104*
Upper lobe	263	51 (19.4)	49 (18.6)**(3; 6.1)**	163 (62.0)**(28; 17.2)**		200	42 (21.0)	99 (49.5)**(6; 6.1)**	59 (29.5)**(25;42.4)**	
Middle lobe	38	7 (18.4)	9 (23.7)**(0)**	22 (57.9) **(0)**		50	25 (50.0)	16 (32.0) **(0)**	9 (18.0) **(1; 11.1)**	
Lower lobe	161	32 (19.9)	22 (13.7)**(1; 4.5)**	107 (66.5)**(7; 6.5)**		150	38 (25.3)	73 (48.7)**(5; 6.8)**	39 (26.0)**(14; 38.5)**	
Not specified	18	5 (27.8)	4 (22.2) **(0)**	9 (50.0)**(1; 11.1)**		13	3 (23.1)	8 (61.5)**(0)**	2 (15.4) **(0)**	
**Border**					*0*.*027*					*0*.*000*
Smooth border or well defined border	127	23 (18.1)	19 (15.0)**(1; 5.3)**	85 (66.9)**(1; 1.2)**		88	26 (29.6)	50 (56.8)**(2; 4.0)**	12 (13.6)**(1; 8.3)**	
Irregular or not well defined										
- Spiculation	50	4 (8.0)	5 (10.0)**(0)**	41 (82.0)**(21; 51.2)**		67	8 (11.9)	24 (35.8)**(7; 29.2)**	35 (52.2)**(20; 57.1)**	
- Lobulation	33	9 (27.3)	3 (9.1)**(0)**	21 (63.6)**(5; 23.8)**		41	8 (19.5)	14 (34.2)**(1; 7.1)**	19 (46.3)**(8; 42.1)**	
- Other irregular	62	11 (17.7)	11 (17.7)**(2; 18.2)**	40 (64.6)**(5; 12.5)**		44	14 (31.8)	20 (45.5)**(0)**	10 (22.7)**(3; 30.0)**	
- Not specified	208	48 (23.1)	46 (22.1)**(1; 2.2)**	114 (54.8)**(4; 3.5)**		173	52 (30.1)	88 (50.9)**(1; 1.1)**	33 (19.1)**(8; 24.2)**	
**Consistency on CT**										*0*.*066*
Solid	-	-	-	*-*		225	54 (24.0)	118 (52.4)**(6; 5.1)**	53 (23.6)**(17; 32.1)**	
Partly solid	-	-	-	*-*		14	3 (21.4)	7 (50.0)**(1;14.3)**	4 (28.6)**(1; 25.0)**	
Ground glass	-	-	-	*-*		25	6 (24.0)	13 (52.0)**(2; 15.4)**	6 (24.0)**(3; 50.0)**	
Calcification	-	-	-	*-*		16	4 (25.0)	11 (68.8) **(0)**	1 (6.3) **(0)**	
Not specified	-	-	-	*-*		133	41 (30.8)	47 (35.3)**(2; 4.3)**	45 (33.8)**(19; 42.2)**	

^(^*^)^ Lung cancer cases are shown in bold for each category together with the risk as a percentage.

According to the data presented in Table 3, immediate interventions were more frequent in patients with nodules larger than 8 mm in both situations; patients with SPN detected in chest radiograph and those detected on CT (218/287, 69.9% and 70/188, 37.2%, respectively) (p<0.001). However, lung cancer diagnosis was more frequently diagnosed in patients with nodules larger than 8 mm who underwent follow-up (32.0% patients with SPN detected in chest radiograph and 36.5% patients with SPN detected in CT) (Table 2).

**Table 3:** Diameter’s gradient according to clinical management strategy (for Chest radiograph and CT) and lung cancer diagnosis after 18 months of follow-up*.

VARIABLES	Chest radiograph					CT				
N (%)										
(95% CI)										
	Total 480 (100%) (40; 8.3%)	No further testing 95 (19.8%) (0)	Follow-up 84 (17.5%) (4; 4.8%)	Immediate interventions 301 (62.7%) (36; 11.9%)	*p-value*	Total 413 (100%) (51; 12.4%)	No further testing 108 (26.2%) (0)	Follow-up 196 (47.5%) (11; 5.6%)	Immediate interventions 109 (26.4%) (40; 36.7%)	*p-value*
**Diameter**					*<0*.*001*					*<0*.*001*
3–4 mm	34	10 (29.4)	13 (38.2)	11 (32.4)		47	14 (29.8)	25 (53.2)	8 (17.0)	
>4–6	74	19 (25.7)	23 (31.1) **(1; 4.3)**	32 (43.2) **(1; 2.5)**		99	39 (39.4)	45 (45.5) **(2, 4.4)**	15 (15.2) **(3, 20.0)**	
>6–8	60	16 (26.7)	21 (35.0)	23 (38.3)		66	16 (24.2)	38 (57.6) **(3, 7.9)**	12 (18.2)	
>8	287	44 (14.1)	25 (8.0) **(8; 32)**	218 (69.9) **(28; 12.8)**		188	33 (17.6)	85 (45.2) **(31, 36.5)**	70 (37.2) **(11, 15.7)**	
Not specified	25	6 (24.0)	2 (8.0)	17 (68.0) **(2; 11.8)**		13	6 (46.2)	3 (23.1)	4 (30.8) **(1, 25.0)**	

^(^*^)^ Lung cancer cases are shown in bold for each category together with the risk as a percentage.

In multivariate analysis, patients with SPN detected on chest radiographs, men were more likely to have immediate interventions (RR 1.45; CI95% 1.23–1.65; p = 0.002). In patients with SPN detected on chest radiographs and CT, spiculated nodules were more likely to have immediate interventions (RR 1.27; CI95% 1.10–1.51; p = 0.034, and RR 3.83; CI95% 2.16–6.79; p<0.001, respectively) and patients with nodules larger than 8 mm were more likely to have immediate interventions (RR 2.35; CI95% 1.43–3.83;p<0.001, and RR 2.19; CI95% 1.13–4.22; p<0.001, respectively).

### Exposure to radiation

In patients undergoing follow-up imaging for SPNs detected on chest radiograph the mean cumulative effective dose was 10.3 mSv. Those ultimately diagnosed with cancer received a significantly higher radiation dose (19.7 vs 9.1, p = 0.027). In those who underwent immediate interventions, the mean cumulative effective dose was 11.6 mSv. Those ultimately diagnosed with cancer received a significantly lower radiation dose (10.4 vs 20.3, p = 0.003). (Table 4).

**Table 4:** Analysis of the radiation exposure (mean, minimum, maximum) associated with the management of SPN for chest radiograph according to the management strategy and for patients with a final diagnosis of lung cancer and those without it.

Intervention	N (%)	Total (mSv)	Cancer (mSv)	No cancer (mSv)	*p-value*
**Follow-up**					
x-ray	35 (41.7)	3.2 (0.2–28.2)	7.2 (7.2–7.2)	3.0 (0.2–28.2)	*0*.*071*
CT	48 (57.1)	15.0 (7.1–60.1)	32.1 (32.1–32.1)	14.2 (7.1–60.1)	*0*.*363*
PET/CT	1 (1.2)	39.1 (39.1–39.1)	-	39.1 (39.1–39.1)	*-*
**Total**	**84 (17.5)**	**10.3 (0.2–60.1)**	**19.7 (7.2–32.1)**	**9.9 (0.2–60.1)**	***0*.*027***
**Immediate intervention**					
x-ray	67 (22.3)	1.7 (0.2–14.2)	-	1.7 (0.2–14.2)	*-*
CT	225 (74.8)	14.0 (7.1–60.1)	19.3 (7.1–39.1)	13.1 (7.1–60.1)	*0*.*099*
PET/CT	9 (3.0)	26.3 (25.1–28.1)	25.9 (25.1–27.1)	26.7 (25.1–28.1)	*0*.*444*
**Total**	**301 (62.7)**	**11.6 (0.2–60.1)**	**10.4 (7.1–39.1)**	**20.3 (0.2–60.1)**	***0*.*003***
**TOTAL**	**480 (100.0)**	**9.1 (0.1–60.1)**	**20.3 (7.1–39.1)**	**8.1 (0.1–60.1)**	***<0*.*001***

In patients undergoing follow-up imaging for SPNs detected on CT the mean cumulative effective dose was 24.4 mSv. There was no difference when stratified by diagnosis of cancer. In those who underwent immediate intervention, the mean cumulative effective dose was 24.5 mSv. Those ultimately diagnosed with cancer received a significantly lower radiation dose (20.2 vs 26.7, p = 0.003) (Table 5).

**Table 5:** Analysis of the radiation exposure (mean, minimum, maximum) associated with the management of SPN for CT according to the management strategy and for patients with a final diagnosis of lung cancer and those without it.

Intervention	N (%)	Total (mSv)	Cancer (mSv)	No cancer (mSv)	*p-value*
**Follow-up**					
- x-ray	18 (9.2)	12.2 (7.1–28.1)	-	12.2 (7.1–28.1)	*-*
- CT	175 (89.3)	25.5 (14.0–81.0)	33.1 (14.0–49.0)	25.0 (14.0–81.0)	*0*.*018*
- PET/CT	3 (1.5)	34.3 (32.0–39.0)	32.0 (32.0–32.0)	36.5 (32.0–39.0)	*0*.*999*
- **Total**	**196 (47.5)**	**24.4 (7.1–81.0)**	**33.0 (14.0–49.0)**	**23.9 (7.1–81.0)**	***0*.*178***
**Immediate intervention**					
- x-ray	3 (2.7)	20.0 (14.0–32.0)	-	20.0 (14.0–32.0)	
- CT	20 (18.3)	18.0 (7.0–53.0)	19.5 (7.0–32.0)	17.8 (7.0–53.0)	*0*.*999*
- PET/CT	47 (43.1)	36.3 (32.0–53.0)	32.0 (32.0–32.0)	38.8 (32.0–53.0)	*<0*.*001*
- Biopsy	39 (35.8)	14.0 (7.0–53.0)	9.2 (7.0–46.0)	18.1 (7.0–53.0)	*0*.*147*
- **Total**	**109 (26.4)**	**24.5 (7.0–53.0)**	**20.2 (7.0–46.0)**	**26.7 (7.0–53.0)**	***<0*.*001***
**TOTAL**	**305 (100.0)**	**19.9 (7.0–81.0)**	**23.1 (7.0–49.0)**	**19.5 (7.0–81.0)**	***0*.*038***

### Average number of imaging tests

In patients undergoing follow-up imaging for SPNs detected on chest radiograph the median number of additional imaging tests was 2.8. There was no difference when stratified by diagnosis of cancer. In those who underwent immediate interventions, the median number of imaging tests was 2.7. In this group, those ultimately diagnosed with cancer underwent a significantly higher number of imaging studies (3.4 vs 2.6, p <0.001). (Table 6).

**Table 6:** Analysis of the number of imaging tests (mean, minimum, maximum) associated with the management of SPN for chest radiograph according to the management strategy and for patients with a final diagnosis of lung cancer and those without it.

Intervention	N (%)	Total	Cancer	No cancer	*p-value*
**Follow-up**					
- x-ray	35 (41.7)	1.4 (1.0–2.0)	1.5 (1.0–2.0)	1.4 (1.0–2.0)	*0*.*733*
- CT	48 (57.1)	1.2 (0–5.0)	1.0 (1.0–1.0)	1.2 (0.0–5.0)	*0*.*736*
- PET/CT	1 (1.2)	0.1 (0–1.0)	0.5 (0–1.0)	0.1 (0.0–1.0)	*<0*.*001*
- **Total**	**84 (17.5)**	**2.8 (2.0–8.0)**	**3.8 (3.0–4.0)**	**2.7 (2.0–8.0)**	***0*.*085***
**Immediate intervention**					
- x-ray	67 (22.3)	1.2 (1.0–2.0)	1.0 (1.0–1.0)	1.3 (1.0–2.0)	*0*.*001*
- CT	225 (74.8)	1.2 (0–6.0)	1.5 (0–4.0)	1.2 (0–6.0)	*0*.*087*
- PET/CT	9 (3.0)	0.1 (0–1.0)	0.3 (0–1.0)	0.1 (0–1.0)	*0*.*001*
- **Total**	**301 (62.7)**	**2.7 (2.0–7.0)**	**3.4 (2.0–5.0)**	**2.6 (2.0–7.0)**	***<0*.*001***

In patients undergoing follow-up imaging for SPNs detected on CT the median number of additional imaging tests was 3.5. Those ultimately diagnosed with cancer underwent a significantly higher number of imaging studies (4.6 vs 3.4, p = 0.009). In those who underwent immediate interventions, the median number of imaging tests was 3.0. In this group, those ultimately diagnosed with cancer underwent a significantly lower number of imaging studies (2.6 vs 3.1, p = = 0.021) (Table 7).

**Table 7:** Analysis of the number of imaging tests (mean, minimum, maximum) associated with the management of SPN for CT according to the management strategy and for patients with a final diagnosis of lung cancer and those without it.

Intervention	N (%)	Total	Cancer	No cancer	*p-value*
**Follow-up**					
- x-ray	18 (9.2)	0.1 (0–1.0)	0	0.1 (0–1.0)	*0*.*280*
- CT	175 (89.3)	3.2 (1.0–8.0)	3.3 (1.0–7.0)	3.2 (1.0–8.0)	*0*.*946*
- PET/CT	3 (1.5)	0.1 (0–1.0)	0.5 (0–1.0)	0.1 (0–1.0)	*<0*.*001*
- **Total**	**196 (47.5)**	**3.5 (2.0–9.0)**	**4.6 (3.0–7.0)**	**3.4 (2.0–9.0)**	***0*.*009***
**Immediate intervention**					
- x-ray	3 (2.7)	0 (0–1.0)	0	0 (0–1.0)	*0*.*212*
- CT	20 (18.3)	1.7 (1.0–5.0)	1.1 (1.0–3.0)	2.0 (1.0–5.0)	*<0*.*001*
- PET/CT	47 (43.1)	0.6 (0–1.0)	0.5 (0–1.0)	0.6 (0–1.0)	*0*.*491*
- **Total**	**109 (26.4)**	**3.0 (2.0–6.0)**	**2.6 (2.0–5.0)**	**3.1 (2.0–6.0)**	***0*.*021***

In S1 and S2 Tables, we show the analysis of the radiation exposure (total) associated with the management of SPN for both chest radiograph and CT according to the management strategy and for patients with a final diagnosis of lung cancer and those without it.

## Discussion

In this study, we describe the different management strategies of SPN when first detected either on chest radiograph or on CT in the usual care setting, their relationship with lung cancer diagnosis and the radiation exposure associated during follow-up of 18 months.

More than 20% of patients with SPN detected on either chest radiograph (19.8%) or CT (26.1%) did not have additional interventions and none of them developed lung cancer (100% negative predictive value). Hence, in contrast with previous studies [13], the patients included in our study did not appear to have received less evaluation than they should. Out of 480 patients with SPN detected on chest radiograph 346 (72.0%), and 254 (61.5%) of the 413 patients with SPN detected on CT, had additional diagnostic tests and were not diagnosed with lung cancer.

Those patients with a SPN initially detected on either chest radiograph or CT, who were followed up, underwent a high number of CTs during the 18 month follow-up period (patients with SPN initially detected on CT underwent a maximum of 8 CTs and those with SPN initially detected on chest radiograph underwent a maximum of 5 CTs). According to Fleischner recommendations, these patients had more CTs than should have been done, conferring more radiation exposure. Previous studies also have presented an excess of invasive procedures in patients with a benign nodule showing that clinicians seem to be unaware that nodule management guidelines exist, or just do not to follow them [15].

Overall, patients with SPN detected on CT received 19.9 mSv radiation exposure per capita during the 18 months of follow-up. Moreover, the maximum radiation dose per capita received during this period of time was 81.0 mSv. Therefore, these results showed a relevant associated radiation exposure, given that the epidemiological data directly suggest an increased cancer risk in the 10 mSv to 100 mSv range [24]. A previous study showed 0.7% incidence of cancer and 1% mortality attributable to imaging tests during a period of 22 years.

The increase in the use of medical imaging in clinical practice [25] fuels concern about radiation exposure and cumulative risk of cancer [26]. In fact, in 2013 the European Union legislation set out a series of directives regarding radiation protection and included the safe use of ionizing radiation in medical practice [27]. One key innovation is the need to record the radiation dose received by each patient undergoing a medical imaging test involving x rays, particularly CT, in order to reduce unnecessary exposure to radiation. Hence, clinicians have a key role in controlling the effective dose received by each patient during the management of SPN and limiting the number of CTs following available guidelines.

However, the increasing availability of machines capable of iterative reconstruction are likely to decrease the radiation exposure significantly. Some authors have demonstrated that low dose scans are acceptable and do not result in changes of nodule volume [28]. Chest radiographs are usually chosen for surveillance in order to limit radiation exposure, and according to these data this approach could lead to low effective doses.

The variables associated with a higher probability of having immediate interventions were for both chest radiograph and CT nodule spiculated borders and size above 8 mm and for chest radiographs also the patients’ sex. Women who had a chest radiograph were less likely to have immediate interventions than men, because lung cancer used to be thought of as a man’s disease, given that the smoking status is more frequent in men than in women. In contrast with previous studies, smoking habit was not a factor influencing immediate interventions. Maybe, the high number of not available data in this variable could explain this fact.

Fleischner recommendations [11], state that although in low-risk patients follow-up ofSPN ≤4 mm is not needed, low- and high-risk patients with SPN >4mm should be followed. In our study, out of 47 patients with SPN ≤4 mm detected on CT, 33 (70.2%) had either follow-up or additional interventions, and none of them were diagnosed of lung cancer (approximately half of them were categorized as low-risk patients –data not shown-). Moreover, 88 patients with nodules >4 mm detected on CT did not have further testing and none of them were diagnosed with lung cancer. When both management and lung cancer diagnosis were analysed for the cut-off 5 mm (according to BTS guidelines [10] and the latest information from the Nelson trial [12]), only 4 (4.4%) patients were diagnosed of lung cancer. Thus, these results could support that the 4mm recommended by Fleischner is probably too low. Our results would confirm the appropriateness of the new established criteria.

The American College of Chest Physicians [16] and the BTS [10] recommended that in patients with SPN >8mm in diameter, clinicians should estimate the pretest probability of malignancy using for example the Brock model [29]. In our study, in those patients who had a CT, only radiographic characteristics (size and border) were associated with a higher probability of having immediate interventions. However, although clinicians did not follow-up the majority of the established recommendations, patients did not seem to receive under-evaluation; in contrast, a significant number of the patients received an excessive amount of interventions when compared to guideline recommendations. Moreover, given the long time until the diagnosis was ascertained in some cases, an effort should be made in the improvement of the quality of care reducing anxiety to patients.

The independent risk factors for malignancy included in the available recommendations are slightly different from those identified in our study [21]. Thus, large series of nodules in low risk population will probably need guidelines adapted to routine clinical care.

Nodule follow-up was heterogeneous and perhaps standardizing the approach to SPN follow up, would be to review these patients in a multidisciplinary meeting including both pulmonologists and radiologists.

Our patients were followed during 18 months due to financial reasons. This could seem short to be certain about the diagnosis in smaller nodules. Since evidence of malignant disease for at least 2 years is a reliable indicator of a benign process [30], some of the patients could have developed the disease in the following years. However, we believe that given the natural history of lung cancer, the management of SPN during 18 months is essential in the prognosis of a patient. Lung cancer could be present without having been diagnosed, but it could be unethical to carry out new diagnostic procedures in patients without a clinical suspicion. Nevertheless, none of the patients were lost at follow-up. The high percentage of non-interventions for CT and chest radiograph could suggest a high number of benign features, which would not reflect the real-world clinical dilemma. However, benign lesions, intrapulmonary lymph and pseudolesions, when detected, were excluded from our study.

## Conclusions

Our results show the different SPN management procedures in the usual care setting. Patients who did not have additional interventions were not diagnosed with lung cancer. However, there was an excessive amount of interventions in a high percentage of patients presenting SPN, which was associated with an excess of radiation exposure.

## Supporting Information

S1 Fig Management strategies carried out in patients having SPN for chest radiography and the detailed description of the diagnostic pathway. (TIF)

S2 Fig Management strategies carried out in patients having SPN for CT and the detailed description of the diagnostic pathway. (TIF)

S1 Table Analysis of the radiation exposure (total) associated with the management of SPN for chest radiograph according to the management strategy and for patients with a final diagnosis of lung cancer and those without it. (DOC)

S2 Table Analysis of the radiation exposure (total) associated with the management of SPN for CT according to the management strategy and for patients with a final diagnosis of lung cancer and those without it. (DOC)

S1 Text Management strategies carried out in patients having SPN for chest radiography and the detailed description of the diagnostic pathway. (DOC)

S2 Text Management strategies carried out in patients having SPN for CT and the detailed description of the diagnostic pathway. (DOCX)
