# Supplementary figures and images for: The Fate of Patients with Solitary Pulmonary Nodules: Clinical Management and Radiation Exposure Associated
Source: PLoS One. 2016 Jul 8;11(7):e0158458. doi: 10.1371/journal.pone.0158458 (PMC4938621; doi:10.1371/journal.pone.0158458)

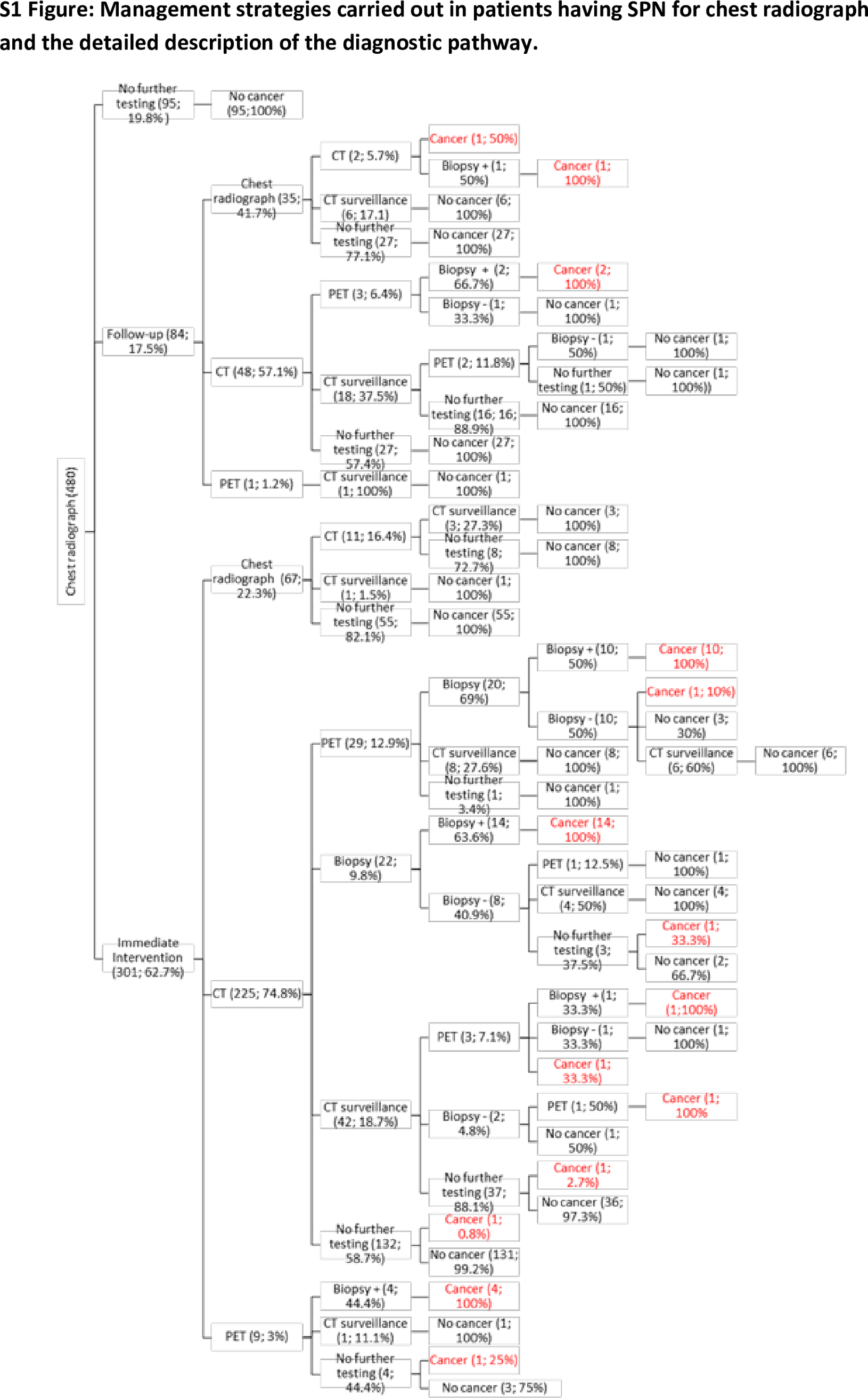

Supplement: S1 Fig — (TIF) [file pone.0158458.s001.tif]

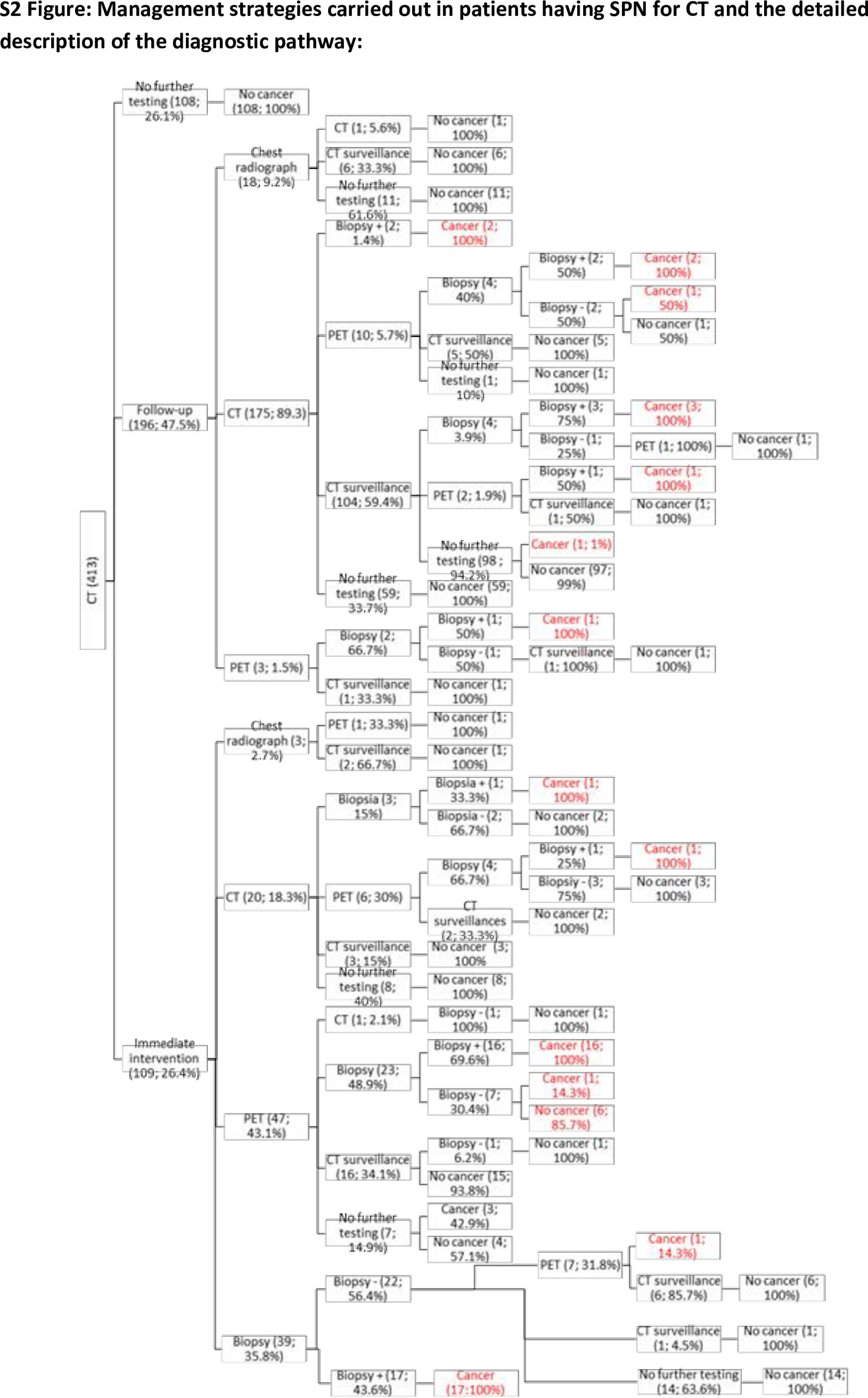

Supplement: S2 Fig — (TIF) [file pone.0158458.s002.tif]
