# Supplementary material for: The Fate of Patients with Solitary Pulmonary Nodules: Clinical Management and Radiation Exposure Associated
Source: PLoS One. 2016 Jul 8;11(7):e0158458. doi: 10.1371/journal.pone.0158458 (PMC4938621; doi:10.1371/journal.pone.0158458)
Supplement: S1 Table — (DOC) [file pone.0158458.s003.doc]

**S1 table: Analysis of the radiation exposure (total) associated with the management of SPN for chest radiograph according to the management strategy and for patients with a final diagnosis of lung cancer and those without it:**

| **Intervention** |  | **N (%)** | **Total (mSv)** | **Cancer (mSv)** | **No cancer (mSv)** |
| --- | --- | --- | --- | --- | --- |
| **Follow-up** |  |  |  |  |  |
|  | x-ray | 35 (41.7) | 112 | 14.4 | 97.6 |
|  | CT | 48 (57.1) | 717.8 | 64.2 | 653.6 |
|  | PET/CT | 1 (1.2) | 39.1 | - | 39.1 |
|  | **Total** | **84 (17.5)** | **868.9** | **78.6** | **790.3** |
|  |  |  |  |  |  |
| **Immediate intervention** |  |  |  |  |  |
|  | x-ray | 67 (22.3) | 114.4 | - | 111.4 |
|  | CT | 225 (74.8) | 598.1 | 598.1 | 2542.4 |
|  | PET/CT | 9 (3.0) | 133.5 | 133.5 | 103.4 |
|  | **Total** | **301 (62.7)** | **2757.2** | **2757.2** | **731.6** |
| **TOTAL** |  | **480 (100.0)** | **810.2** | **810.2** | **3556.9** |
