# Supplementary material for: The Fate of Patients with Solitary Pulmonary Nodules: Clinical Management and Radiation Exposure Associated
Source: PLoS One. 2016 Jul 8;11(7):e0158458. doi: 10.1371/journal.pone.0158458 (PMC4938621; doi:10.1371/journal.pone.0158458)
Supplement: S2 Table — (DOC) [file pone.0158458.s004.doc]

**S2 table: Analysis of the radiation exposure (total) associated with the management of SPN for CT according to the management strategy and for patients with a final diagnosis of lung cancer and those without it:**

| **Intervention** |  | **N (%)** | **Total (mSv)** | **Cancer (mSv)** | **No cancer (mSv)** |
| --- | --- | --- | --- | --- | --- |
| **Follow-up** |  |  |  |  |  |
|  | x-ray | 18 (9.2) | 218.8 | - | 218.8 |
|  | CT | 175 (89.3) | 4462.0 | 331.0 | 4131.0 |
|  | PET/CT | 3 (1.5) | 103.0 | 32.0 | 71.0 |
|  | **Total** | **196 (47.5)** | **4783.8** | **363.0** | **4420.8** |
|  |  |  |  |  |  |
| **Immediate intervention** |  |  |  |  |  |
|  | x-ray | 3 (2.7) | 60.0 | - | 60.0 |
|  | CT | 20 (18.3) | 360.0 | 39.0 | 321.0 |
|  | PET/CT | 47 (43.1) | 1707.0 | 544.0 | 11663.0 |
|  | Biopsy | 39 (35.8) | 546.0 | 165.0 | 381.0 |
|  | **Total** | **109 (26.4)** | **2673.0** | **748.0** | **1925.0** |
| **TOTAL** |  | **305 (100.0)** | **8212.8** | **1111.0** | **7101.8** |
