# Supplementary material for: The Fate of Patients with Solitary Pulmonary Nodules: Clinical Management and Radiation Exposure Associated
Source: PLoS One. 2016 Jul 8;11(7):e0158458. doi: 10.1371/journal.pone.0158458 (PMC4938621; doi:10.1371/journal.pone.0158458)
Supplement: S1 Text — (DOC) [file pone.0158458.s005.doc]

**S1 Text: Management strategies carried out in patients having SPN for chest radiograph and the detailed description of the diagnostic pathway:**

We initially classified the diagnostic pathways into:

1) *No further testing*: Out of 480 patients with SPN, 95 (19.8%) were not testing further and none of them were diagnosed of lung cancer.

2) *Follow-up three months after the detection of SPN*: 84 (17.5%) patients were followed up and 4 (4.7%) patients were diagnosed of lung cancer (median time to diagnosis: 9 months; IQR 7-11 months). The additional imaging tests carried out were:

o Chest radiograph: 35 (41.7%) patients were initially followed-up with chest radiograph: 27 (77.1%) patients did not undergo further testing; 6 (17.1%) received CT surveillance (CT average number 2.18; standard deviation 0.98) and 2 (5.7%) patients received 1 CT and/or biopsy and were diagnosed of lung cancer (median time to diagnosis: 13 months; IQR 10- 16 months).

o CT: 48 (57.1%) patients were initially followed-up with CT: 27 (57.4%) of them were not testing further; 3 (6.4%) received PET/CT (2 patients were diagnosed of lung cancer; median time to diagnosis: 7 months; IQR 6-8 months), and 18 (37.5%) received CT surveillance and/or PET/CT (test average number 2.00; standard deviation 0.91).

o PET/CT: 1 (1.2%) patient was initially followed-up with PET/CT and CT surveillance (1 follow-up CT 6 months after), who was not diagnosed of lung cancer.

3) *Immediate intervention (during the three months after the detection of SPN):* 301 (62.7%) patients had immediate interventions and 36 (11.9%) patients were diagnosed of lung cancer (median time to diagnosis: 2 months; IQR 1-4 months). The additional imaging tests carried out were:

o Chest radiograph: 67 (22.3%) patients had chest radiograph and none of them were diagnosed of lung cancer: 55 (82.1%) did not undergo further testing; 11 (16.4%) had 1 CT and 3(27.3%) of them received CT surveillance (1 CT more), and 1 (1.5%) had CT surveillance (1 follow-up CT 12 months after).

o CT: 225 (78.4%) patients had a CT:

1. 29 (12.9%) had PET/CT: 20 (69%) of them had biopsy and 11 (55%) were diagnosed of lung cancer (median time to diagnosis: 2 months; IQR 1-3 months); 8 (27.6%) received CT surveillance (test average number 1.5; standard deviation 1.07), and 1 (3.4%) was not testing further.
2. 22 (9.8%) had biopsy: 14 (63.6%) had a positive biopsy (median time to diagnosis: 2 months; IQR 1-3 months), and 8 had a negative biopsy. Of these: 1 (12.5%) patient had PET/CT; 4 (50%) had CT surveillance (test average number 1.5; standard deviation 0.57), and 3 (37.5%) did not undergo further testing, where 1 patient was finally diagnosed of lung cancer (time to diagnosis: 2 months).
3. 42 (18.7%) had CT surveillance (test average number 1.67; standard deviation 0.93): 3 (7.1%) had PET/CT and 2 patients were diagnosed of lung cancer (median time to diagnosis: 13 months; IQR 11-15 months); 2 (4.8%) had biopsy and/or PET/CT and 1 (50%) was diagnosed of lung cancer (time to diagnosis, 5 months), and 37 (88.1%) were not testing further and 1 (2.7%) was diagnosed of lung cancer (time to diagnosis, 4 months).
4. 132 (58.7%) did not undergo further testing and 1 (0.8%) was diagnosed of lung cancer (time to diagnosis, 1 month).

o PET/CT: 9 (3%) patients had a PET/CT and 5 (55.6%) patients were diagnosed of lung cancer (median time to diagnosis: 1 month; IQR 0.5-1.3 months):

1. 4 (44.4%) had a positive biopsy (median time to diagnosis: 1 month; IQR 0.5-1.3 months).
2. 1 (11.1%) had CT surveillance (3 followed CT at 3, 6 and 12 months after).
3. 4 (44.4%) were not testing further and 1 (25%) was diagnosed of lung cancer (median time to diagnosis not available).
