# Supplementary material for: The Fate of Patients with Solitary Pulmonary Nodules: Clinical Management and Radiation Exposure Associated
Source: PLoS One. 2016 Jul 8;11(7):e0158458. doi: 10.1371/journal.pone.0158458 (PMC4938621; doi:10.1371/journal.pone.0158458)
Supplement: S2 Text — (DOCX) [file pone.0158458.s006.docx]

**S2 Text: Management strategies carried out in patients having SPN for CT and the detailed description of the diagnostic pathway:**

We initially classified the diagnostic pathways into:

1. *No further testing*: Out of 413 patients with SPN, 108 (26.1%) patients did not undergo further testing and none of them were diagnosed of lung cancer.
2. *Follow-up three months after the detection of SPN*: 196 (47.5%) patients were followed up and 11 (5.6%) patients were diagnosed of lung cancer (median time to diagnosis: 7 months; IQR 6-12 months). The additional imaging tests carried out were:
   1. Chest radiograph: 18 (9.2%) patients were initially followed-up with chest radiograph and none of them were diagnosed of lung cancer: 11 (61.6%) were not testing further; 6 (33.3%) had CT surveillance (test average number 2.00; standard deviation 0.89) and 1 (5.6%) received 1 follow-up CT.
   2. CT: 175 (89.3%) patients were initially followed-up with CT and 8 (4.5%) were diagnosed of lung cancer:
      1. 2 (1.4%) had a positive biopsy (median time to diagnosis: 15 months; IQR 10-20 months).
      2. 10 (5.7%) had PET/CT: 4 (40%) had biopsy, where 2 patients had a positive biopsy (median time to diagnosis: 5 months; IQR 2-7 months) and out 2 patients with negative biopsy, 1 was diagnosed of lung cancer (time to diagnosis 7 months); 5 (50%) had CT surveillance (test average number 2.88; standard deviation 0.44), and 1 (10%) did not undergo further testing.
      3. 104 (59.4%) had CT surveillance (test average number 2.14; standard deviation 0.92): 4 (3.9%) had biopsy and 3 (75%) were diagnosed of cancer (median time to diagnosis: 17 months; IQR 16-18 months); 2 (1.9%) had PET/CT, where 1 of them (50%) had positive biopsy (time to diagnosis 9 months) and 1 (50%) had CT surveillance (4 follow-up CT), and 98 did not undergo further testing (1 patient was diagnosed of lung cancer, median time to diagnosis 10 months).
      4. 59 (33.7%) were not testing further.
   3. PET/CT: 3 (1.5%) patients were initially followed-up with PET/CT: 2 (66.7%) had biopsy, where 1 of them was diagnosed of lung cancer (time to diagnosis 3 months) and 1 (33.3%) had CT surveillance (4 follow-up CT).
3. *Immediate intervention (during the three months after the detection of SPN):* 109 (26.4%) patients had immediate interventions and 40 (36.7%) patients were diagnosed of lung cancer (median time to diagnosis: 0.8 months; IQR 0.3-1.5 months). The additional imaging tests carried out were:

- Chest radiograph: 3 (2.7%) patients had a chest radiograph and none of them were diagnosed of lung cancer: 1 (33.3%) had PET/CT and (66.7%) had CT surveillance (test average number 2.50; standard deviation 0.71).
- CT: 20 (18.3%) patients had a CT: 3 (15%) had biopsy, where 1 patient was diagnosed of lung cancer (time to diagnosis 1.5 months); 6 (30%) had PET/CT (4 (66.7%) of the patients had biopsy, where 1 patient was diagnosed of lung cancer - time to diagnosis 0.4 months-, and 2(33.3%) had CT surveillance -test average number 1.00; standard deviation 0); 3 (15%) had CT surveillance (test average number 2.00; standard deviation 1.00), and 8 (40%) were not testing further.
- PET/CT: 47 (43.1%) patients had PET/CT: 1 (2.1%) had CT and a negative biopsy; 23 (48.9%) underwent biopsy and 17 of them (73.9%) were diagnosed of lung cancer (median time to diagnosis: 0.8 months; IQR 0.4-2.0 months); 16 (34.1%) underwent CT surveillance (test average number 1.75; standard deviation 0.77), and 7 (14.9%) were not testing further (3; 42.9%, were diagnosed of lung cancer, median time to diagnosis: 0.7 months; IQR 0.5-1.9 months).
- Biopsy: 39 (35.8%) patients had a biopsy: 17 (43.6%) had positive biopsy (median time to diagnosis: 0.7 months; IQR 0.2-0.9 months) and 22 (56.4%) had negative biopsy (1 patient was diagnosed of lung cancer).
